# Phaeohyphomycosis in China

**DOI:** 10.3389/fcimb.2022.895329

**Published:** 2022-06-13

**Authors:** Yun He, Hai-lin Zheng, Huan Mei, Gui-xia Lv, Wei-da Liu, Xiao-fang Li

**Affiliations:** ^1^ Institute of Dermatology, Chinese Academy of Medical Science and Peking Union Medical College, Nanjing, China; ^2^ Institute of Dermatology, Chinese Academy of Medical Science, Jiangsu Key Laboratory of Molecular Biology for Skin Diseases and STIs, Nanjing, China; ^3^ Skin Disease Prevention and Treatment Institute of Yixing, Yixing, China

**Keywords:** phaeohyphomycosis, melanized fungi, epidemiology, risk factors, clinical features, diagnosis, treatment strategy, drug sensitive

## Abstract

**Background:**

Due to more attentions paid to melanized fungi over the past few decades and under the background of the global coronavirus disease 2019 pandemic (COVID-19) the fact that the virus itself and the immunosuppressive agents such as glucocorticoids can further increase the risk of infections of deep mycoses, the number of patients with phaeohyphomycosis (PHM) has a substantial increase. Their spectrum is broad and the early diagnosis and treatments are extremely sticky. This study aims to more comprehensively understand the clinical features of phaeohyphomycosis in China over 35 years and to establish a more applicable systematical classification and severity grades of lesions to guide treatments and prognosis.

**Methods:**

We reviewed 174 cases of proven phaeohyphomycosis reported in Chinese and English language literature from 1987 to 2021 and we also made the accurate classification definitions and detailed information about the epidemiology, species of clinical dematiaceous fungi, minimum inhibitory concentration values, clinical features, treatments, and prognosis.

**Results:**

The mortality of cerebral, disseminated and pulmonary phaeohyphomycosis are 55%, 36%, and 25%. Nearly 19% of patients had poor quality of life caused by the complications such as disability, disfigurements, and blindness. The overall misdiagnosis rate of phaeohyphomycosis was 74%. Moderate to severe rashes are accounting for 82% of subcutaneous phaeohyphomycosis. The areas of the head and face are mostly affected accounting for 16% of severe rashes. Nearly 30% of invasive infections of phaeohyphomycosis are triggered by recurrent lesions. Voriconazole, itraconazole, amphotericin B deoxycholate (AmB-DOC), and terbinafine were most commonly used but diagnosis and treatments of phaeohyphomycosis remain challenging in reality.

**Conclusions:**

Our classifications are likely to be more practical and easier to popularize, and there are still also plenty of characteristics in these non-specific lesions. There’re no significant variations in cure rates, or death rates between three grades of lesions. But patients with severe rashes have longer courses and lower effective rates.

## Introduction

Phaeohyphomycosis is a group of mycoses caused by pigmented fungi characterized by yeast-like cells, hyphae, or a combination of these in tissues. When it comes to PHM, firstly it should be distinguished from the primary implantation mycoses caused by melanized fungi including eumycetoma and chromoblastomycosis (CBM). Eumycetoma, involves deep musculoskeletal tissue, characterized by the presence of granules and sclerotia in sinus secretions (McGinnis, 1983; Revankar and Sutton, 2010). CBM, involves cutaneous tissue, characterized by the muriform cells on histopathology (McGinnis, 1983; Revankar and Sutton, 2010; Queiroz-Telles et al., 2017). Some melanized fungi may cause allergic diseases, but they were extremely rarely reported and would not be further discussed.

PHM is an opportunistic infection that has been reported in patients scattered around the world, which is prevalent in tropical and subtropical areas of the planet (Revankar et al., 2002; Revankar et al., 2004; Revankar and Sutton, 2010). More than 150 species and 70 genera of clinical pathogenic fungi of PHM have been found (Revankar and Sutton, 2010). These melanized fungi affect different hosts and cause diverse syndromes in organisms with different propensities ranging from the superficial to deep tissues which include central nervous system (CNS) infections, disseminated infections, pulmonary infections, deep-local infections, subcutaneous infections, and superficial infections. PHM has been recently found related closely to some immune deficiencies which may be natural, especially the deficiency of the caspase recruitment domain-containing protein 9 (CARD9) gene or acquired (Revankar and Sutton, 2010). Increasingly more attention has been paid to these fungi ever since the past few decades especially under the background of the COVID-19 pandemic when the immunosuppressive agents such as glucocorticoids were widely used. The number of reported patients with PHM has a substantial increase recently (Latawa et al., 2022; Borman et al., 2022). More critically, PHM is often misdiagnosed at an early stage and the treatment remains intractable. At present, there is lack of detailed information about almost all types of PHM from China during 35 years covering the scopes of clinical features, treatments, and prognosis based on a large sample, which also provides more applicable systematic classifications and the severity grads of lesions. To provide more practical information about Chinese experiences in PHM for extensive clinical workers to achieve early diagnosis and better management of this sophisticated mycosis, and therefore, we collected and analyzed the whole attainable case reports of PHM in China from 1987 to 2021.

## Methods

### Literature Search

We searched for English resources from PubMed, Google Scholar, and Embase. At the same time, we also searched for Chinese resources from China National Knowledge Internet (CNKI), WanFang and WeiPu. We used the strategy “(China OR Taiwan OR Hong Kong OR Macao) and (phaeohyphomycosis OR black OR dematiaceous OR melanized OR phaeoid)” and the genus names of known clinical major dematiaceous fungi. Cases reported from 1987 to 2021were enrolled.

### Inclusion and Exclusion Criteria

Criteria for study inclusion:

1. direct microscopy or histopathology showing dark septate hyphae and/or yeast-like elements from invaded tissue or
2. culture or polymerase chain reaction confirming a melanized fungus from clinical specimen and
3. compatible clinical syndrome

Criteria for study exclusion:

1. repeated reports and patients out of the territory of China and
2. patients diagnosed with chromoblastomycosis or mycetoma and
3. patients diagnosed with allergic disease caused by dematiaceous fungi.

### Classification

Refer to the most authoritative reviews published by Sanjay G. Revankar and Flavio Queiroz Telles in 2002, 2004, 2010, and 2017, cases were classified as (Revankar et al., 2002; Revankar et al., 2004; Revankar and Sutton, 2010; Queiroz-Telles et al., 2017):

1. CNS phaeohyphomycosis was defined as cases of primary cerebral infections including brain abscess, meningitis, encephalitis, myelitis, and meningoencephalitis; clinical specimens mainly consist of brain lesions tissues or cerebrospinal fluid (CSF).
2. disseminated PHM were defined as cases with the recovery of the isolate from blood samples or evidence of infections at 2 noncontiguous sites;
3. pulmonary PHM were defined as cases of primary pulmonary infections including pneumonia, asymptomatic solitary pulmonary nodule, and endobronchial lesion; clinical specimens mainly consist of lung or bronchial lesion tissues, sputum, and bronchoalveolar lavage fluid (BALF);
4. deep local PHM were defined as cases of primary local-deep infections including endophthalmitis, invasive rhinosinusitis, and miscellaneous infections; clinical specimens mainly consist of deep local lesion tissues and drainage fluids;
5. subcutaneous PHM were defined as cases of primary subcutaneous infections mainly including nodular cyst, solid papules, and verrucous plaques; clinical specimens mainly consist of either skin lesion tissues or purulent aspirates;
6. keratitis PHM were defined as cases of primary corneal infections; clinical specimens mainly consist of corneal scrapings;
7. superficial PHM were defined as cases of primary superficial infections including onychomycosis, tinea nigra, cutaneous and mucosal infections; clinical specimens mainly consist of skin or nail scrapings, and mucosal swabs.

### Statistical Analysis

We calculated descriptive statistics (frequencies, means, medians) for demographic, clinical, and, laboratory variables. We applied chi-square analysis with Fisher’s exact test to compare groups if needed. Excel and SPSS were used in data collection and analysis. P-value ≤ 0.05 is statistically significant.

## Results

163 relevant literature were collected, among which 174 patients from 142 articles met the inclusion criteria.

### Epidemiology

**Figure 1:**
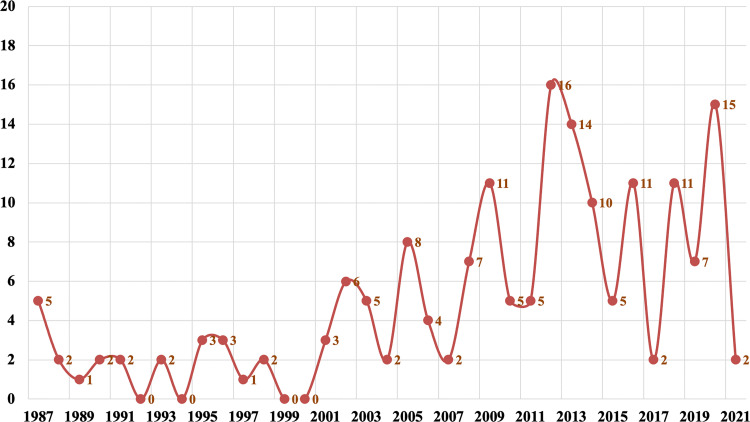
The number of reported cases of PHM in China.

**Figure 2:**
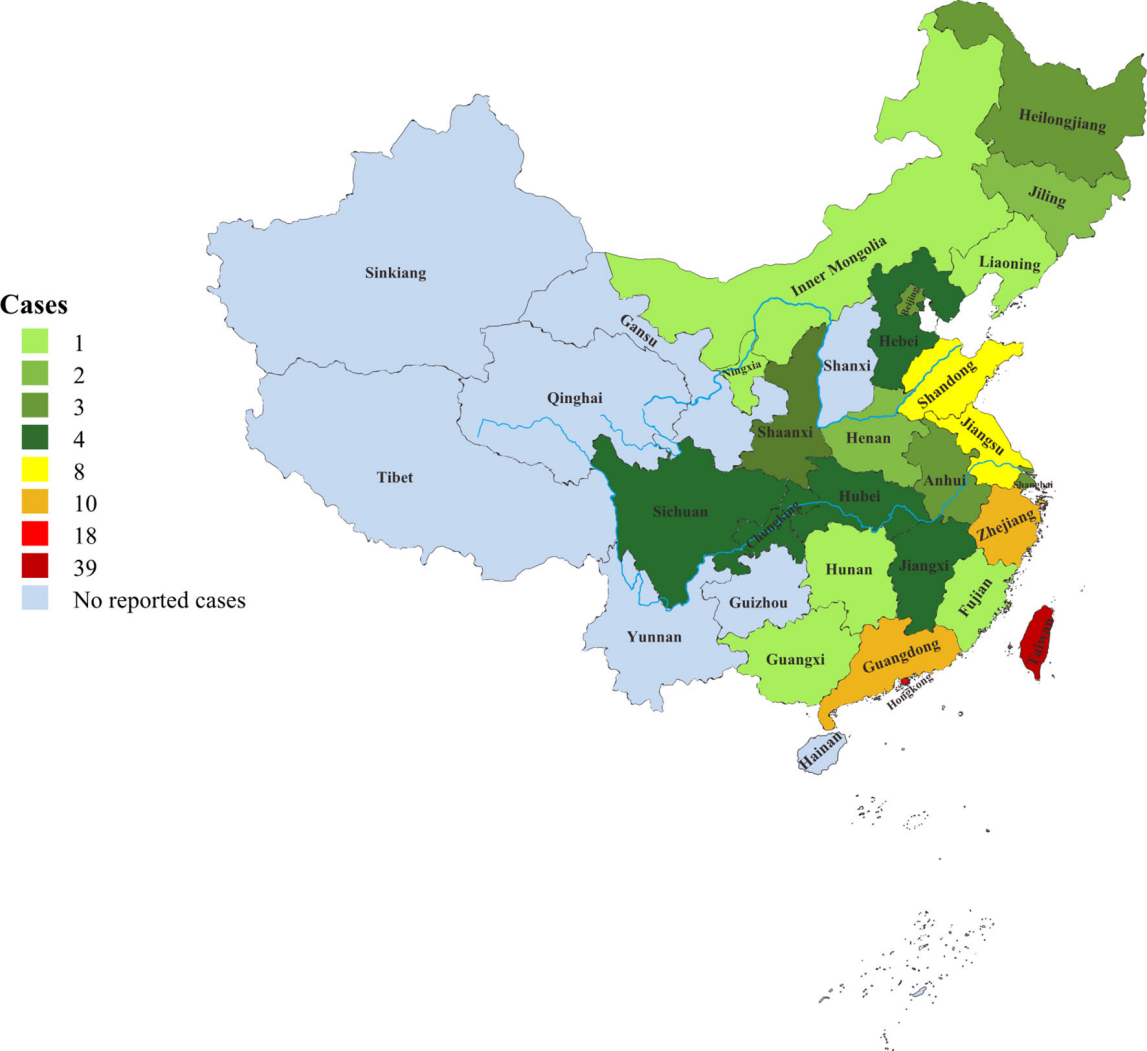
The distribution patterns of PHM in China.

Since 2000, there had been a rapid growth in the number of reported phaeohyphomycosis in China. The number of cases in the last 20 years was about 6.6 times that of 20 years ago (**Figure 1**). There were 118 (68%) patients from the mainland, 39 (22%) patients from Taiwan, and 17 (10%) patients from Hong Kong. Most cases were sporadic in tropical and subtropical regions, although some cases were in temperate regions, mainly between latitudes of 3°N and 53°N. Most patients came from southern China, including Taiwan, Zhejiang, Jiangsu, and Guangdong provinces (**Figure 2**).

### Demographics and Risk Factors

A total of 174 patients were enrolled in this study, including 107 males and 67 females. The ratio of male to female patients was 1·6:1. The mean age at diagnosis was 48 years (range, 2–89 years; median age, 53years). The most common risk factors for phaeohyphomycosis infections were traumas (37%), diabetes (11%), and corticosteroid use (11%). Inherited CARD9 deficiency (7%) was found in either severe refractory or mild PHM infections. Malnutrition (6%), tumors (5%), kidney transplantations (3%), and chemotherapy (2%) were frequent risk factors in patients with disseminated, CNS, deep local, and pulmonary infections. Another 22% of the patients did not find any obvious risk factors (**Table 1**).

**Table 1:** Demographics and risk factors of phaeohyphomycosis in China.

Infection type	Total	CNS	Disseminated	Pulmonary	Deep-local	Subcutaneous	Keratitis	Superficial
**Demographics (Ratio %)**	n=174	n=11	n=11	n=8	n=10	n=85	n=26	n=23
**Male**	107 (61%)	11 (100%)	5 (45%)	7 (88%)	6 (60%)	49 (58%)	16 (62%)	13 (57%)
Female	67 (39%)	0 (100%)	6 (55%)	1 (13%)	4 (40%)	36 (42%)	10 (38%)	10 (43%)
Age, mean (year)	48	29	26	54	61	51	57	37
Range (year)	2-89	4-73	9-56	10-89	45-75	2-89	22-79	2-87
Risk factor ** (Ratio %)**								
**Stem cell transplantation**	0 (0%)	0 (0%)	0 (0%)	0 (0%)	0 (0%)	0 (0%)	0 (0%)	0 (0%)
Heart transplantation	1 (1%)	0 (0%)	0 (0%)	0 (0%)	0 (0%)	1 (1%)	0 (0%)	0 (0%)
Lung transplantation	0 (0%)	0 (0%)	0 (0%)	0 (0%)	0 (0%)	0 (0%)	0 (0%)	0 (0%)
Liver transplantation	2 (1%)	0 (0%)	0 (0%)	1 (13%)	1 (10%)	0 (0%)	0 (0%)	0 (0%)
Kidney transplantation	5 (3%)	0 (0%)	1 (9%)	1 (13%)	0 (0%)	3 (4%)	0 (0%)	0 (0%)
Graft vs host disease	1 (1%)	0 (0%)	1 (9%)	0 (0%)	0 (0%)	0 (0%)	0 (0%)	0 (0%)
Corticosteroid use	20 (11%)	0 (0%)	1 (9%)	1 (13%)	1 (10%)	14 (16%)	0 (0%)	3 (13%)
Other immunosuppressants	9 (5%)	0 (0%)	0 (0%)	1 (13%)	1 (10%)	5 (6%)	0 (0%)	2 (9%)
Malignancy	9 (5%)	1 (9%)	1 (9%)	0 (0%)	2 (20%)	4 (5%)	1 (4%)	0 (0%)
Chemotherapy	3 (2%)	0 (0%)	1 (9%)	0 (0%)	1 (10%)	0 (0%)	1 (4%)	0 (0%)
Neutropenia	2 (1%)	0 (0%)	0 (0%)	1 (13%)	1 (10%)	0 (0%)	0 (0%)	0 (0%)
HIV/AIDS	0 (0%)	0 (0%)	0 (0%)	0 (0%)	0 (0%)	0 (0%)	0 (0%)	0 (0%)
**Primary T-cell immunodeficiency**	1 (1%)	0 (0%)	0 (0%)	0 (0%)	0 (0%)	1 (1%)	0 (0%)	0 (0%)
CARD9 mutation	12 (7%)	1 (9%)	2 (18%)	0 (0%)	0 (0%)	9 (11%)	0 (0%)	0 (0%)
Malnutrition	10 (6%)	0 (0%)	2 (18%)	1 (13%)	1 (10%)	6 (7%)	0 (0%)	0 (0%)
Pregnancy	3 (2%)	0 (0%)	1 (9%)	0 (0%)	0 (0%)	2 (2%)	0 (0%)	0 (0%)
Trauma	65 (37%)	2 (18%)	5 (45%)	0 (0%)	4 (40%)	25 (29%)	22 (85%)	7 (30%)
Smoke	2 (1%)	0 (0%)	1 (9%)	1 (13%)	0 (0%)	0 (0%)	0 (0%)	0 (0%)
Diabetes mellitus	20 (11%)	2 (18%)	1 (9%)	0 (0%)	4 (40%)	12 (14%)	0 (0%)	1 (4%)
Chronic liver disease	2 (1%)	0 (0%)	0 (0%)	0 (0%)	0 (0%)	1 (1%)	1 (4%)	0 (0%)
Chronic pulmonary disease	11 (69%)	0 (0%)	1 (9%)	2 (25%)	0 (0%)	8 (9%)	0 (0%)	0 (0%)
Chronic renal disease	4 (2%)	0 (0%)	0 (0%)	0 (0%)	1 (10%)	3 (3%)	0 (0%)	0 (0%)
Chronic heart disease	3 (2%)	0 (0%)	0 (0%)	0 (0%)	0 (0%)	3 (3%)	0 (0%)	0 (0%)
No risk factor	38 (22%)	3 (27%)	0 (0%)	2 (25%)	1 (10%)	19 (22%)	2 (8%)	11 (48%)

### Clinical Classification and Features

In 2017, Flavio Queiroz Telles et al. proposed the spectrum of fungal diseases caused by melanized fungi in order from superficial to deep (Queiroz-Telles et al., 2017). Based on their spectrum of diseases, we reclassified the types of phaeohyphomycosis. The mortality rate was prioritized. Cerebral infections were ranked first. Disseminated infections were ranked second and pulmonary infections were ranked third. Given the highest incidence of subcutaneous and corneal infections, they were separated from the deep-local infection types, and therefore seven applicable subclasses were divided. The spectrum of clinical syndromes was summarized as follows (**Table 2**):

- CNS Infections
- Disseminated Infections
- Pulmonary Infections
- Other Deep Local Infections

▲ Eumycetoma (not included)

Subcutaneous Infections

▲ Chromoblastomycosis (not included)

Keratitis
Superficial Infections

**Table 2:** Infection types of the phaeohyphomycosis in China.

Infection types	Subtypes	Total (Ratio%)	Reference
central nervous system infection	–	11 (6%)	(Wang et al., 1991; Lirng et al., 1995; Lv et al., 2001; Chang et al., 2009; Huang et al., 2011; He, 2012; Chen et al., 2013; Hu et al., 2014; Tong et al., 2020; Bai et al., 2020; Lai et al., 2021)
disseminated infection	–	11 (6%)	(Dai et al., 1987; Wan et al., 1987; Li, 1988; Jiang and Zhang, 1991; Wang et al., 2003; Tseng et al., 2005; Dong et al., 2009; Zhang et al., 2017; Yao, 2018; Wang et al., 2019; Zhang et al., 2019)
pulmonary infection	–	8 (5%)	(Zhao et al., 2002; Woo et al., 2008; Xu et al., 2010; To et al., 2012; Woo et al., 2013; Ye et al., 2014; Wang et al., 2016; Liu et al., 2018)
deep local infection	endophthalmitis	3 (2%)	(Jin et al., 1996; Sun et al., 2010; Liu et al., 2015)
	acute invasive rhinosinusitis	1 (1%)	(Fang et al., 2011)
	hepatic infection	1 (1%)	(Tsang et al., 2021)
	bone and joint infection	2 (1%)	(To et al., 2012; Wang et al., 2014)
	peritonitis	1 (1%)	(Lau et al., 2003)
	pleuritis	1 (1%)	(Liu et al., 1997)
	lymphnoditis	1 (1%)	(Liou et al., 2002)
subcutaneous infection		85 (49%)	(Zheng et al., 1988; Wang et al., 1989; Lv, 1990; Zhao et al., 1990; Zhang et al., 1990; Hsu and Lee, 1993; Lin et al., 1995; Chuan and Wu, 1995; Li et al., 1996; Zhao et al., 2001; Liou et al., 2002; Chen et al., 2003; Matsushita et al., 2003; Yang et al., 2004; Lv et al., 2005; Xia et al., 2005; You et al., 2005; Li et al., 2005; Yu et al., 2006; Hang et al., 2006; Chen et al., 2006; Chen et al., 2008; Liu and Wu, 2008; Huang et al., 2008; Fan et al., 2009; Zhou et al., 2009; Lin et al., 2009; Jin et al., 2009; Zeng et al., 2010; Lin et al., 2010; Zhang et al., 2010; Lv et al., 2011; Sang et al., 2011; Sang et al., 2012; Dong et al., 2012; Zhou et al., 2012; Lin et al., 2012; Li et al., 2012; Ge et al., 2012; Woo et al., 2013; Hsiao et al., 2013; Cai et al., 2013; Liu et al., 2013; Chen et al., 2014; Wang et al., 2014; Tsang et al., 2014; Tsang et al., 2014; Hsu et al., 2015; Hu et al., 2015; Zhang and Lian, 2015; Wang et al., 2015; Chen et al., 2016; Zhou et al., 2016; Chen et al., 2016; Hu et al., 2016; Ng et al., 2017; Ge et al., 2017; Zhou et al., 2018; Wang et al., 2018; Xie et al., 2018; Feng et al., 2018; Yang et al., 2018; Yang et al., 2019; Liu et al., 2019; Wang et al., 2019; Huang et al., 2019; Guo et al., 2019; Gong et al., 2020; Linqiang et al., 2020; Chen et al., 2021; Yu et al., 2021; Pan et al., 2021)
keratitis		26 (15%)	(Huang et al., 1998; Liu et al., 2002; Zhu and Xu, 2003; Tsai et al., 2006; Zhang et al., 2006; Cao and Wu, 2008; Li et al., 2009; Yang et al., 2009; Liu et al., 2011; Lu et al., 2012; Qiu and Yao, 2013; Wang et al., 2016; Zhong et al., 2007; Hung et al., 2020)
superficial infection	tinea nigra	10 (6%)	(Gu et al., 1996; Liu et al., 2001; Dai and Zhang, 2004; Wu et al., 2005; Pi et al., 2005; Qu et al., 2007; Wang et al., 2008; Tan et al., 2014; Lu et al., 2016; Guo et al., 2016)
	cutaneous	4 (2%)	(Hsu and Lee, 1993; Li et al., 2008; Woo et al., 2013; Chen et al., 2016)
	onychomycosis	9 (5%)	(Yu et al., 2009; Huang et al., 2009; Sun and Ju, 2011; Woo et al., 2013; Wen et al., 2016; Shi et al., 2016)

#### A. CNS Infections

A total of 10 patients were included. Brain abscess was the most common presentation, seen in 6 cases, 3 cases of only brain abscess, and 3 cases combined with meningitis. In addition, there were 2 cases of encephalitis, 2 cases of myelitis, and 2 cases of meningoencephalitis. The early clinical manifestations were fever, headache, intracranial hypertension, change in consciousness and behavior, hemiplegia, and defect in the visual field. Changes in consciousness and behavior included anxiety, retardation, coma, convulsions, *etc.* Hemiplegia was usually manifested as unilateral limb weakness. The late stage of CNS infections presented with extensive ventricular obstruction, cerebral infarction hydrocephalus, and cerebral hernia, leading to central respiratory failure eventually.

#### B. Disseminated Infections

A total of 11 patients were included. The most common manifestations were fever and swelling of superficial lymph nodes. Fevers were seen in 6 cases and swollen lymph nodes were seen in 5 cases. The most common original sites of disseminated infections included skins (4), lungs (2), brain (1), lymph node (1), pharynx (1), eye (1), and blood (1). The initial skin manifestations included generalized black warty patches (2) and dark facial erythemas (2). Respiratory and CNS complaints were commonly seen in 3 cases with clinical signs of cough with sputum, dyspnea, and hemiplegia left. Four patients’ original infections came from the eyes, pharynx, and face finally spreading to the center in the advanced disease stages. One patient’s pulmonary infection eventually progressed to fatal sepsis. All five patients died. The positive blood cultures were seen in 3 patients and blood was the only site of infection in one patient. All infected sites by dematiaceous fungi were ranged in order from the commonest to the least common, including skin (6), brain (5), lymph node (4), bone (4), blood (3), lung (3), sinus (2), pharynx (2), oral (1), eye (1), muscle (1), liver (1), gall (1) and spleen (1). The mean number of organs involved was 3 per patient (range, 1–7 organs).

#### C. Pulmonary Infections

A total of 8 patients were included. The clinical manifestations included pneumonia (5), asymptomatic pulmonary nodules (2), and endobronchial lesions (2). The commonest early symptoms were fever, cough with sputum, and exacerbation to empiric antibiotic therapies. The number of melanized fungi pneumonia added up to 5 progressing rapidly to respiratory failure, hypoxemia, and sepsis in short term. Three of these patients died of multiple organ failures due to uncontrolled fungal pneumonia.

#### D. Other Deep Local Infections

##### a. Endophthalmitis

Three patients were included. Palpebral tuberosity was presented in one patient. A progressive corneal ulcer to endophthalmitis was seen in the other two cases. The initial symptoms had foreign body sensations and impaired visions. Later there would be increased intraocular pressures and intense pains.

##### b. Acute Invasive Rhinosinusitis

One patient was included. Left nasal obstruction with yellow discharge and burning pain from the face to the head were the initial symptoms. Left eyelid ptosis, impaired vision, and exophthalmos occurred for half a month. Progressive peripheral facial paralysis combined with vision and hearing loss was seen in the late stage of the infection, which resulted in severe Rhino-Orbito-Brain Infection Syndrome.

##### c. Miscellaneous Infections

Two patients with bone and joint infections mainly presented suppurative arthritis of the right knees, progressive painful swellings, and joint fluids. One patient with hepatic infection had liver abscesses and progressive abdominal pain. Lymphnoditis was seen in one patient with two palpable lymph nodes in the left axilla (2 x 2 cm and 1.5 x 1 cm). Peritonitis in a single case in which the clinical features included diarrhea, progressive aggravated periumbilical pain, tenderness, and rebound pain. Pleuritis in one patient had the manifestation of progressive aggravated right thoracalgia with massive pleural effusion.

#### E. Subcutaneous Infections

**Figure 3:**
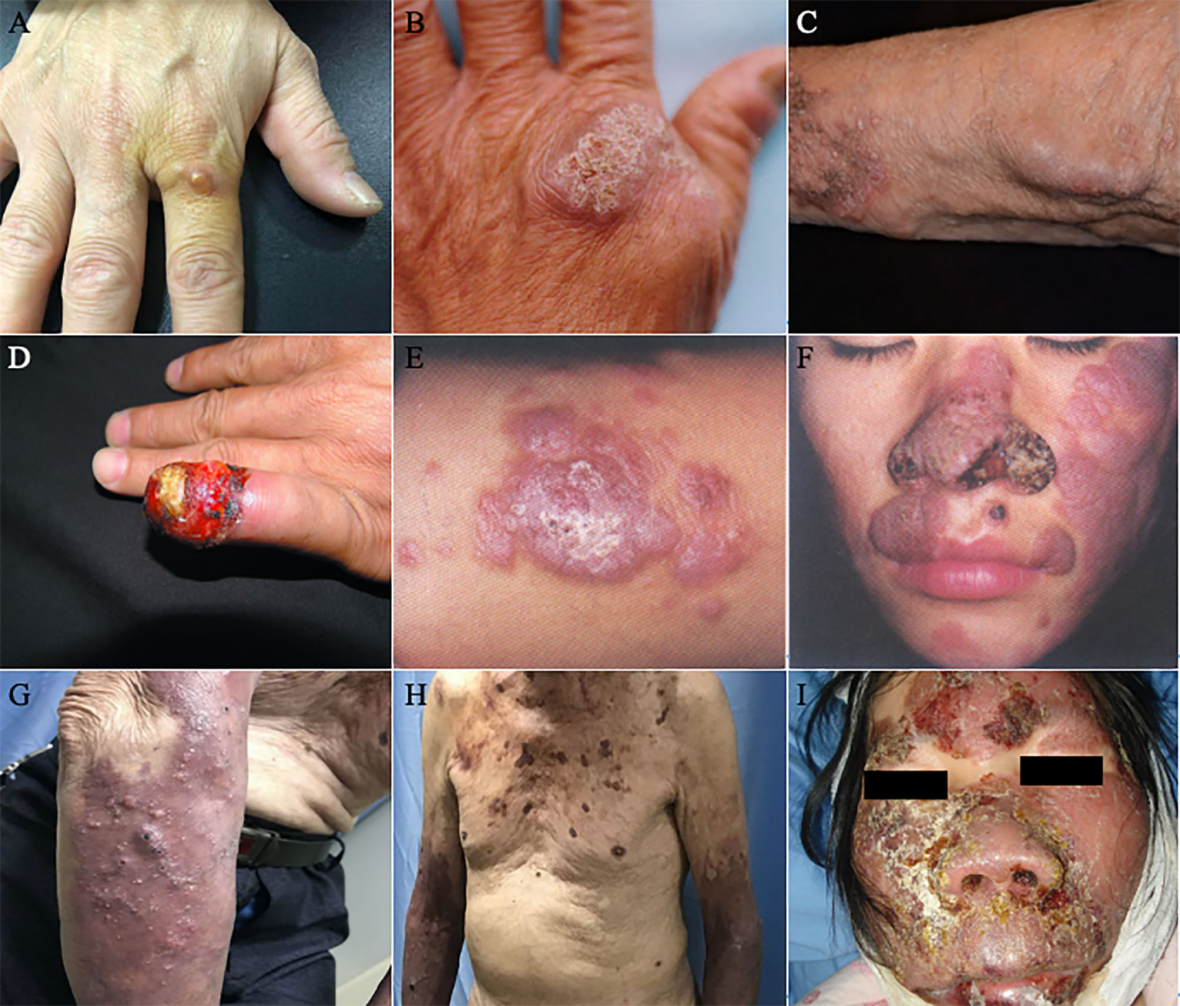
Clinical types of lesions observed in patients with PHM. **(A)** Glossy papules lesions on the right hand. (Reproduced from reference Linqiang et al. (2020) [original **Figure 1B**]) **(B)** Yellowish verrucous plaques on the left hand. (Reproduced from reference Chen et al. (2008) [original **Figure 1A**]) **(C)** Sporotrichoid nodules lesions on the left forearm. (Reproduced from reference Yu et al. (2021) [original **Figure 1D**]) **(D)** Swelling erythematous erosions and necrosis on the left little finger. (Reproduced from reference Lin et al. (2009) [original **Figure 1**]) **(E)** Infiltrative erythematous plaques surrounded by little papules on the left forearm. (Reproduced from reference Wang et al. (2004) [original **Figure 2**]) **(F)** Infiltrating red plaques on both sides of the face and the upper lip; disfiguring verrucous plaques and ulcers on either side of the nasal ala. (Reproduced from reference Wang et al., (2004) [original **Figure 1**]) **(G) (H)** Nodules, papules, hemorrhagic vesicles, and pustules lesions on the bilateral forearm and trunk. (Reproduced from reference Pan et al. (2021) [original **Figure 1A, B**]) **(I)** Infiltrative swelling erythematous plaques on the face with purulent, smelly discharge. (Reproduced from reference Yan et al. (2016) [original **Figure 1A**]).

**Figure 4:**
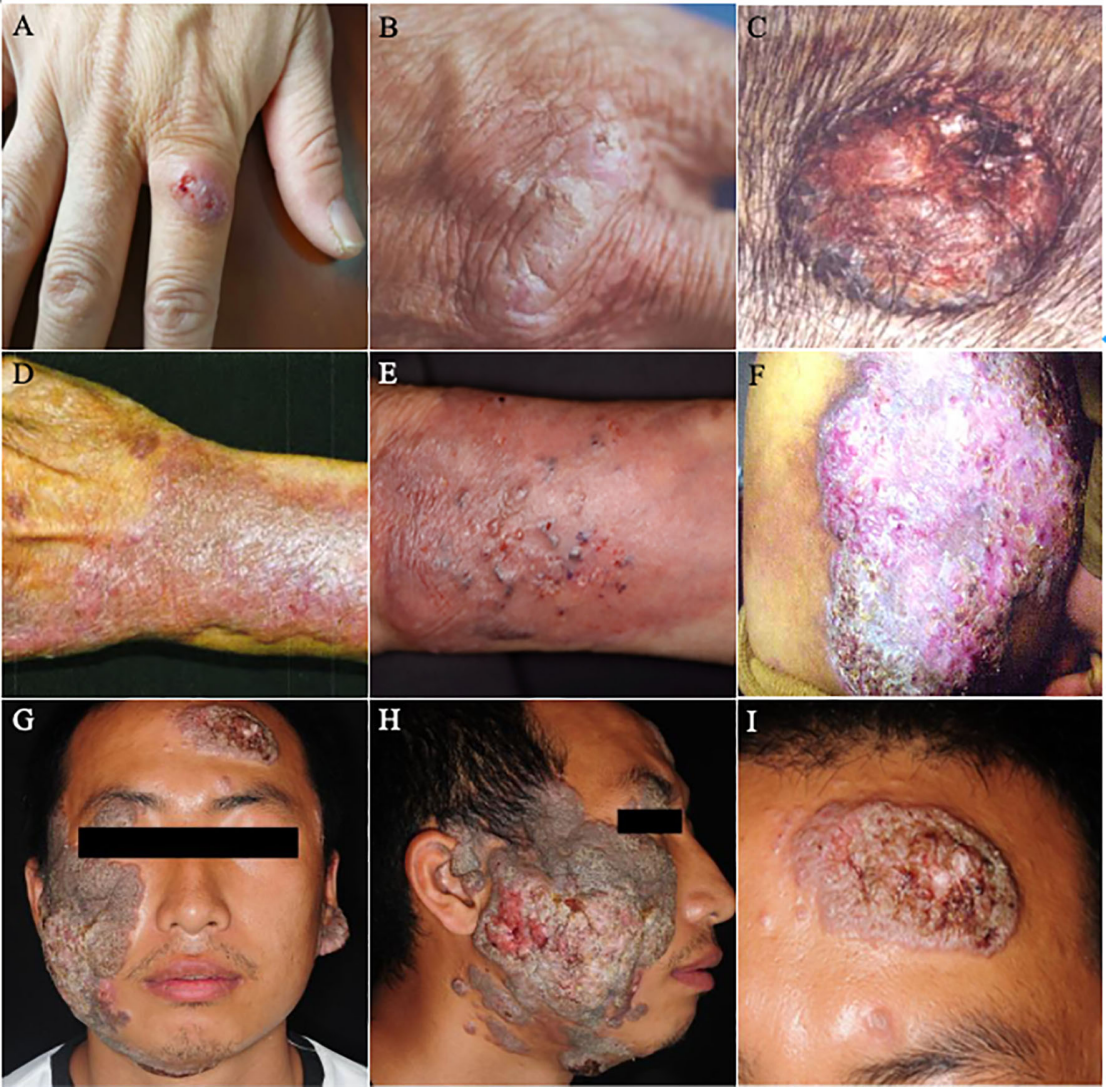
Lesions of PHM with different severity grades. **(A–C)** Mild forms (**(A)**: Reproduced from reference Linqiang et al. (2020) [original **Figure 1A**]); **(B)**: Reproduced from reference Huang et al. (2008) [original **Figure 1**]; **(C)**: Reproduced from reference Lin et al. (2010) [original **Figure 1A**]); **(D–F)** Moderate forms (**(D)**: Reproduced from reference Liu et al. (2013) [original **Figure 1**]; **(E)**: Reproduced from reference Yu et al. (2021) [original **Figure 1G**]; **(F)**: Reproduced from reference Lv et al. (2005) [original **Figure 1**]); **(G–I)** Severe forms (Reproduced from reference Huang et al. (2019) [original Figure **A–C**]).

A total of 85 patients were included. Referred to the authoritative review of Flavio Queiroz Telles, subcutaneous lesions were classified into three grades (**Table 3**, **Table 4**, **Figure 3**, **Figure 4**) (Revankar and Sutton, 2010). Moderate to severe rashes are the most common accounting for 82% (70 of 85). Infiltrating plaque (72%, 28 of 39) and solid or cystic nodules (44%, 17 of 39) were the most common in moderate rashes. Verrucous plaques in severe rashes (45%, 14 of 31) were more common. Mild lesions were dominated by single plaque and nodules (67%, 10 of 15). Secondary lesions mostly included crusts, purulence, and ulcers. Vesicular, purpura, and sinus were less common. Mild and moderate lesions were mostly seen on the arms and legs, while severe lesions mostly occurred on the areas of the head, face, and upper limbs. In the late stages of infections, the invasions of the nasal mucosa, eyes, pharynx, muscles, bones, and lymphatics were not rare (26%, 8 of 31). About 36% of disseminated types of phaeohyphomycosis in China developed from chronic and recurrent rashes. The chief complaints of these patients were pain (12%, 10 of 85) and itching (25%, 21 of 85). There’re no significant differences in either curative rates (P=2.06) or death rates (P=2.57) among mild, moderate, and severe rashes. They are approaching a level of significant differences in effective rates and the average treatment courses among the three kinds of rashes. The data showed that the more severe the rash is, the lower the effective rates (P=0.08) and the longer average treatment course (125,134 and 239 days respectively, P=0.098) will be. We concluded that the severity of lesions is directly related to the prognosis of the disease and the immune state, which may predict the patient’s clinical outcome.

**Table 3:** Clinical severity gradation and criteria classification in patients with subcutaneous PHM.

Severity Grade	Classification Criteria
Mild	a single plaque, nodule, or eschar with a diameter<5 cm
Moderate	single or multiple lesions such as nodules, plaques, or verrucous occurring alone or in combination, covering one or two adjacent skin areas, with a diameter between 5–15 cm
Severe	any type of skin lesion alone or in combination covering extensive body areas or invasion of subcutaneous fat, muscle, bone, or other adjacent tissue and not identified as the disseminated infection

**Table 4:** Severity gradation and clinical manifestation in patients with subcutaneous PHM.

Severity of disease	Mild	Moderate	Severe	Total
	n=15	n=39	n=31	n=85
Diameter				
≤5cm	15 (100%)	0 (0%)	0 (0%)	15 (18%)
>5cm and ≤15cm	0 (0%)	39 (100%)	2 (6%)	41 (48%)
>15cm	0 (0%)	0 (0%)	29 (94%)	29 (34%)
Adjacent Tissue Invasion				
Fat	0 (0%)	0 (0%)	2 (6%)	2 (2%)
Muscle	0 (0%)	0 (0%)	2 (6%)	2 (2%)
Nasal Mucosa	0 (0%)	0 (0%)	1 (3%)	1 (1%)
Maxilla	0 (0%)	0 (0%)	1 (3%)	1 (1%)
Type of Lesion				
Papule	1 (7%)	5 (13%)	9 (29%)	15 (18%)
Nodule	5 (33%)	17 (44%)	16 (52%)	38 (45%)
Plaque	5 (33%)	28 (72%)	21 (68%)	54 (64%)
Verruca	2 (13%)	10 (26%)	14 (45%)	26 (31%)
Tumor	3 (20%)	1 (3%)	3 (10%)	7 (8%)
Swelling	0 (0%)	7 (18%)	4 (13%)	11 (13%)
Vesicle	0 (0%)	0 (0%)	1 (3%)	1 (1%)
Purpura	0 (0%)	2 (5%)	2 (6%)	4 (5%)
Purulence	2 (13%)	23 (59%)	12 (39%)	37 (44%)
Black Dot	0 (0%)	3 (8%)	2 (6%)	5 (6%)
Errhysis	0 (0%)	0 (0%)	5 (16%)	5 (6%)
Ulceration	1 (7%)	17 (44%)	11 (35%)	29 (34%)
Necosis	0 (0%)	2 (5%)	2 (6%)	4 (5%)
Crust	4 (27%)	21 (54%)	17 (55%)	42 (49%)
Cicatricial	0 (0%)	3 (8%)	7 (23%)	10 (12%)
Sinus	0 (0%)	0 (0%)	1 (3%)	1 (1%)
Infected Body Part				
One Body Part				
Head and neck	3 (20%)	10 (26%)	5 (16%)	18 (21%)
Upper limb	8 (53%)	14 (36%)	4 (13%)	26 (31%)
Lower limb	4 (27%)	12 (31%)	3 (10%)	19 (22%)
Trunk	0 (0%)	0 (0%)	1 (3%)	1 (1%)
Bottock	0 (0%)	0 (0%)	2 (6%)	2 (2%)
Two Body Parts				
Head and neck+Upper limb	0 (0%)	1 (3%)	2 (6%)	3 (4%)
Head and neck+Trunk	0 (0%)	0 (0%)	2 (6%)	2 (2%)
Head and neck+Buttock	0 (0%)	0 (0%)	1 (3%)	1 (1%)
Upper limb+Lower limb	0 (0%)	0 (0%)	3 (10%)	3 (4%)
Upper limb+Trunk	0 (0%)	0 (0%)	1 (3%)	1 (1%)
Three Body Parts or More	0 (0%)	0 (0%)	7 (23%)	7 (8%)
Symptom				
Pain	2 (13%)	5 (13%)	3 (10%)	10 (12%)
Itchy	3 (20%)	9 (23%)	9 (29%)	21 (25%)

#### F. Keratitis

A total of 26 patients were involved. The main manifestations were inflammatory infiltrations of the corneal epithelium. They began as well-defined grayish-white or brown patches and then progressed to deep ulcers, which could be accompanied by anterior chamber inflammatory exudations, empyemas, and pupil deformations. The clinical manifestations mainly included foreign body sensations, blurred visions, photophobia tears, and swelling pains. Moreover, empirical antibiotic therapy often failed. In the known data there were 14 cases of right eyes and 7 cases of left eyes. There were two cases of central types, one case of paracentral type, and one case of peripheral type. In addition, seven ulcers were spanning from the peripheral to the paracentral corneas. The available data on sizes of the corneal ulcers were large (>6mm) in 2 cases, medium (2-6mm) in 6 cases, and small (<2mm) in 2 cases.

#### G. Superficial Infections

##### a. Onychomycosis

Nine patients were included. The first toenail was the preferred site in the disease. The nails majorly developed turbid and thickened with debris. And then the infected nails cracked, exfoliated, and destroyed. The colors of nails were potentially from white-yellow to black-brown.

##### b. Tinea Nigra

Ten patients were included. Lesions were characterized by the round, banded, or irregular black patches with scaling occurring on the palms and anterior thorax.

##### c. Cutaneous Lesion

Four patients were included. Dark-brown macules and papules were found on the lower jaw, crus, and pedis. One patient was accompanied by interphalangeal macerations, erosions, and rhagades on the left foot.

### Diagnosis

Fast diagnosis is often critical for the survival of fatal invasive fungal infections, especially crucial for the drugs of systemic phaeohyphomycosis as the usually poor outcome of treatment. The European Society of Clinical Microbiology and Infectious Diseases (ESCMID) and European Confederation of Medical Mycology (ECMM) joint clinical guidelines recommended the diagnosis of systemic PHM in 2014 including histopathology, culture, and sequencing for definitive species identification (Chowdhary et al., 2014).

**Figure 5:**
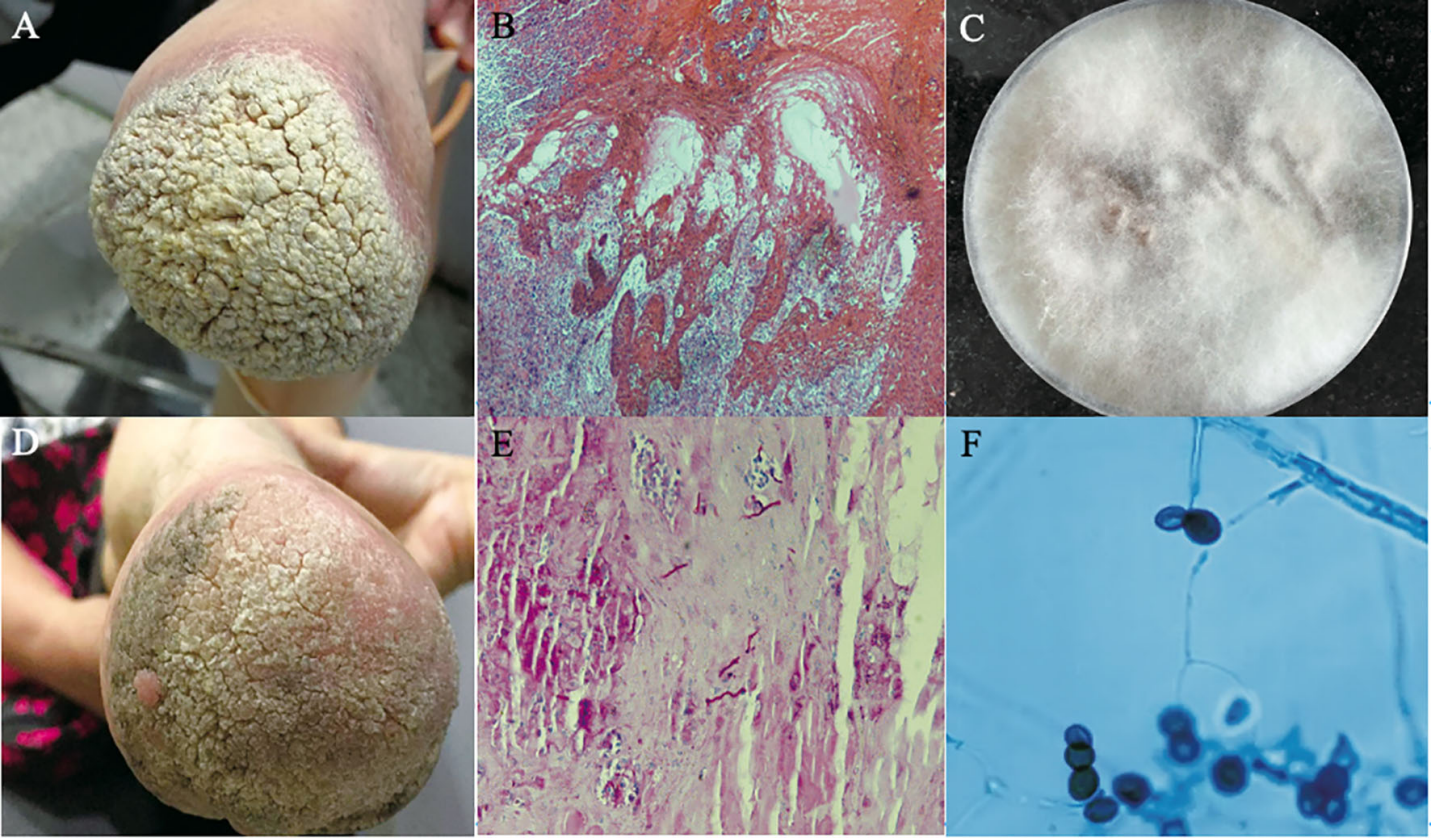
Diagnosis of a case of PHM caused by *Arthrinium phaeospermum*. **(A)** Hypertrophic verrucous plaques with basal infiltrating erythema at the distal left lower extremity in a 59-year-old female with a history of double limb amputations for 20 years; **(B)** Resolved lesions upon follow up after one month; **(C)** Hematoxylin-eosin **(HE)** stained tissue sections showed papillomatous hyperplasia of the spinous layer, mild interspinous cell edema, vascular hyperplasia of the dermal papillary layer, scattered dotted cell infiltration and collagen fiber hyperplasia (HE × 40); **(D)** Periodic acid-Schiff (PAS) stained tissue sections showed scattered hyphae on the epidermis (PAS × 100); **(E)** Colonies on sabouraud dextrose agar (SDA) at 26°C for 2 weeks were hairy, velvety and greyish-white on the front and orange on the back; **(F)** Structures under microculture on potato dextrose agar (PDA) showed that transparent conidia grew from calabash type mother cells and produced lateral conidia. The lens of the conidia eye was black and brown, with a linear bud splitting in middle latitude (lactophenol-cotton blue stain × 400); (Reproduced from reference Zhou et al. (2016) [original **Figure 1**–6]).

Traditionally, the diagnosis of PHM requires a combination of the patient’s clinical manifestations and mycological confirmations (**Figure 5**). The most specific and persuasive diagnostic elements are yellowish-brown hyphae with or without budding cells in skin scrapings, stained tissue slices, aspirated pus, or surgical drainage (Chowdhary et al., 2014). The evidence can be obtained directly by KOH microscopic examination, pathology, fungal culture, or animal models. When the infections are superficial or on the corneal, diagnosis can be made by direct microscopy, and biopsy is not needed. However, pathological biopsy remains the gold standard when diagnosing cerebral, pulmonary, and deep-local infections, as it is not uncommon that the early-stage CSF, BALF and drainage fluid examinations generally show negative fungal cultures or smears. In our study, pieces of evidence of fungal elements were found in 88% (153 of 174) cases that underwent pathological examinations. In subcutaneous infections, pathological diagnosis is based on the clinical manifestations of PHM lesions (Queiroz-Telles et al., 2017). Microscopically, the nodular cyst mainly has palisading epithelioid macrophages at the inner edge of the abscess. The lumen of the abscess often contains necrotic debris mixed with polymorphonuclear leukocytes and the mycelium is prominently seen in the wall of the abscess. The solid papules revealed a thickening epidermis near the hair follicles. Dermal edema, capillary dilation with inflammatory cell infiltration, and acanthosis with granuloma formation are common. Dark fungal elements can be seen within giant cells in the tissue. The old verrucous plaques show hyperkeratosis, parakeratosis, and acanthosis. Lymphocytes, histiocytes, plasma cells, and giant cells are always surrounded by a thick zone of fibroblasts and collagen. The fungal cells are present as yeastlike cells in short chains or toruloid hyphae. The detection rate of fungal elements can be improved in pathologic examination by special staining. Hematoxylin and eosin stain (H&E) stain are the most used, Fontana-Masson stain and Gomori methenamine silver (GMS) stain are sensitive for the detection of fungal cells when fungal elements are scarce (Yan et al., 2019).

However, different species of dark molds cannot be distinguished by pathological sections alone. That’s why we emphasize the necessity of simultaneous culture. Isolation on common sabouraud dextrose agar (SDA) is feasible. It is recommended to turn to plant-based media such as potato dextrose agar (PDA), corn, and oat dextrose agar due to the poor sporulation in part of dark fungi. Most pathogens can develop visible colonies during 1–2 weeks from the initial pale white to the dark colony. The specimens ought to be incubated at 25° to 30°C for 4 weeks before being discarded as negative. Nearly 99% (173 of 174) cases in China obtained dark fungi strains through cultures. Morphological identifications were done in 167 cases. However, confirmation of phenotypic characteristics highly depends on experienced specialists and needs a long cycle. And its limitation of identification to the genus level makes it impossible to provide an early definitive specific diagnosis.

Conservative culture methods have demerits and cannot offer reliable and rapid information to achieve optimal disease management. Sequence analysis of the internal transcribed spacer (ITS) and D1/D2 regions of rDNA are recommended for effective and accurate molecular identification of dark fungi, among which the ITS region has been widely regarded as the universal barcode for the classification of dark fungi. Nearly 54% (94 of 174) cases in China obtained definitive species based on sequence analysis combined with morphological characteristics except for one patient directly through ITS sequencing without fungal culture. It confirms the theory that molecular identification directly from clinical samples is another alternative approach for species identification. The molecular approach especially nuclear ITS sequencing was the most used, accounting for 85% (80 of 94). In addition, 5.8s rDNA, 18s rDNA, 26s rDNA, 28s rDNA, β-tubulin, β-actin, Chitin synthase, Plasma membrane ATPase, (glyceraldehyde-3-phosphate dehydrogenase) *gpd*, (translation elongation factor 1-alpha) *TEF*, (RNA polymerase II largest subunit) *RPB1*, and (the second largest subunit of RNA polymerase II) *RPB2* were used in combination with ITS sequences to verify inter/intra specific variations and to identify new species.

Although the molecular methods are faster than controversial techniques, they have higher cost and are susceptible to contamination due to multi-step processes. Sometimes DNA fragments encoding melanin may inhibit PCR amplification and the limited existing GenBank database may bring difficulty in identifying dark fungi strains (Paul et al., 2017). Around 2010, a technology named MALDI-TOF MS was introduced into the clinical microbiology territory as a more effective and faster diagnostic technique than DNA sequencing (Lau, 2021). It would take 5-9 days for sequence identification of melanized fungi whereas it would take shorter times about 3-7 days for MALDI-TOF MS (Paul et al., 2019). There was also one strain identified as *Phialemonium* species by the use of matrix-assisted laser desorption ionization time–of–flight mass spectrometry (MALDI-TOF MS). With the continuous improvement of the database in the future, this technology will become mature and more widely used for dark mold identification.

In our study, there were a total of 55 dark fungi strains that caused PHM in China (**Supplementary file Table 2**). The most common species of strains were *Exophiala* spp. (33%, 58 of 174) Twelve strains were first reported to cause PHM in the world (**Table 5**). The species identification in China mainly depends on a combination of phenotypic and morphologic features. And also, we found plenty of points of differences in clinical pathogenic strains between China and foreign countries (**Table 6**).

**Table 5:** Twelve strains first reported to cause phaeohyphomycosis in the world.

First Auther (Reference)	Year	Region	Sex/age	Subtype	Genus
Chen Qiuxia (Hang et al., 2006)	2008	Guangdong	M/43 y	subcutaneous	*Cladosporium sphaerospermum*
Dong Mingli (Lu et al., 2016)	2008	NA	M/77 y	subcutaneous	*Knufia epidermidis*
Dong Mingli (Wang et al., 2003)	2009	Jiangsu	F/20 y	CNS	*Exophiala asiatica*
Lv Guixia (Lin et al., 2010)	2011	NA	M/57 y	subcutaneous	*Corynespora cassiicola*
Ge Yiping (Li et al., 2012)	2012	Zhejiang	M/7 y	subcutaneous	*Ochroconis tshawytschae*
Patrick C Y Woo (Xu et al., 2010)	2013	Hong Kong	F/68 y	onychomycosis	*Exophiala hongkongensis*
Chi-Ching Tsang (Liu et al., 2013)	2014	Hong Kong	M/55 y	subcutaneous	*Phialemoniopsis hongkongensis*
Chi-Ching Tsang (Wang et al., 2014)	2014	Hong Kong	M/74 y	subcutaneous	*Hongkongmyces pedis*
Wang Luxia (Lu et al., 2012)	2015	Guangdong	M/54 y	corneal	*Bipolaris oryzae*
Guo Yanyang (Gong et al., 2020)	2019	Shaanxi	F/19 y	subcutaneous	*Pallidocercospora crystallina*
Deng Linqiang (Chen et al., 2021)	2020	Jiangxi	F/45 y	subcutaneous	*Hongkongmyces snookiorum*
Chi-Ching Tsang (Liu et al., 2015)	2021	Hong Kong	M/65 y	Liver	*Pleurostoma hongkongense*

F, female; M, male; NA, not available

**Table 6:** The differences in strains in PHM between China and foreign countries.

Infection Types	Domestic (No., ratio%)	Foreign (Revankar and Sutton, 2010)
CNS	*Exophiala dermatitidis* (4, 36%)*; Cladophialophora bantiana* (2, 18%).	*Cladophialophora bantiana; Rhinocladiella mackenziei; Ochroconis gallopava*
Disseminated	*Exophiala dermatitidis* (3, 27%)*; Exophiala spinifera* (2, 18%)	*Scedosporium apiospermum; Exophiala dermatitidis; Exophiala oligosperma*
Pulmonary	*Chaetomium* spp (2, 25%)*; Exophiala* spp (2, 25%).	*Scedosporium prolificans; Cladophialophora bantiana*
Subcutaneous	*Exophiala* spp (25, 29%)*; Cladosporium* spp (6, 7%)*; Phialophora* spp (5, 6%)*; Veronaea* spp (5, 6%)*; Alternaria* spp (4, 5%)*; Arthrinium* spp (4, 5%)*; Corynespora* spp (4, 5%).	*Stenella araguata;Phoma eupyrena;Chaetomium globosum*
Keratitis	*Colletotrichum* spp (8, 31%); *Chaetomium* spp (5, 19%); *Curvularia* spp (5, 19%); *Exserohilum* spp (3, 12%).	*Curvularia* spp.*;Bipolaris* spp.*;Exserohilum spp*
Tinea nigra	*Hortaea werneckii* (10, 100%)	*Stenella araguata; Phoma eupyrena; Chaetomium globosum*

### Differential Diagnosis

Clinically, clinical presentations of phaeohyphomycosis are particularly non-specific in the early stages. They can mimic a variety of infectious and other diseases (**Table 7**). It is super important to distinguish PHM from a big variety of other confusable diseases as early as possible.

**Table 7:** Main differential diagnoses of phaeohyphomycosis in China.

	Associated Disease(s)
**Infectious, Agent**	
**Fungi**	***Cryptococcus*** (cerebral cryptococcosis and cryptococcosis cutis); **Melanized fungi** (chromoblastomycosis and eumycetoma); ***Sporothrix*** (sporotrichosis cutis); ***Zygomycota*** (rhinofacial zygomycosis)
**Bacteria**	**Bacterium** (cerebral abscess, liver abscess, pneumonia, pleurisy, chronic purulent nasosinusitis, impetigo, and cellulitis); **Tuberculosis** (pulmonary tuberculosis, osseous tuberculosis, orbital tuberculosis, proliferative lymph tuberculosis, and lupus vulgaris); **Cutibacterium acnes** (facial acne)
**Viruses**	**HPV** (verruca vulgaris and plane warts); **HSV** (herpes simplex); **EV70** (acute hemorrhagic conjunctivitis)
**Noninfectious**	
**Tumors**	**Malignancy** (cerebral malignant tumors, lung squamous cell carcinoma, orbital malignant reticulohistiocytoma, thoracolumbar metastasis, skin squamous cell carcinoma); **Benign tumor** (sebaceous gland hyperplasia)
**Others**	discoid lupus erythematosus, pyogenic granuloma, sarcoidosis, tophus, allergic cutaneous vasculitis, lichen planus, fixed drug eruption, pigmentary purpuric, and eczema

HPV, human papilloma virus; HSV, herpes simplex virus; EV70, enterovirus type 70.

### Treatment and Outcome

In this study, we have made a detailed framework to link the treatment with the prognosis of the phaeohyphomycosis patients in China (**Table 8**, **Supplementary Table 3**). The main antifungal agents used in cerebral PHM were triazoles plus amphotericin B deoxycholate (AmB-DOC)/liposomal amphotericin B (L-AmB), among triazoles voriconazole was the most used. Cerebral cysts were usually combined with surgeries. The disseminated infections mainly used triazoles plus AmB-DOC/L-AmB, terbinafine, or 5–fluorocytosine (5-FC). Voriconazole, itraconazole, fluconazole, and ketoconazole all had been tried. Most pulmonary infections were initially treated with combination therapy, including caspofungin plus AmB-DOC or voriconazole, and maintained with oral itraconazole or voriconazole in the stable phases. Deep-local infections were mainly used AmB-DOC plus voriconazole or itraconazole. Most mild subcutaneous infections and keratitis were initially treated with triazoles, including itraconazole, voriconazole, and posaconazole. Severe subcutaneous mainly chose triazoles combined with AmB-DOC and terbinafine. Terbinafine is lipophilic and widely distributed in organizations, especially in the stratum corneum, which has been mostly used for subcutaneous and superficial infections. But as we counted, triazoles are still the dominant antifungal agents in skin infections. Itraconazole was given in doses of 200mg to 400mg daily, voriconazole in doses of 100mg to 800mg daily, and terbinafine in doses of 250mg to 500mg daily. All corneal infections had resorted to keratoplasty when drug therapies were not effective. Among echinocandins, only caspofungin was used in pulmonary infections, mainly in combination with voriconazole and AmB-DOC. Posaconazole, a new triazole, was infrequently used and was tried as a single agent in one patient with cerebral PHM and another patient with severe subcutaneous PHM. The median treatment duration of all PHM in China was 60 days (range, 1–4745days).

**Table 8:** Therapy for phaeohyphomycosis in 174 patients in China.

No. (Ratio%)	Total	CNS	Disseminated	Pulmonary	Deep-local	Subcutaneous	Keratitis	Superficial
Therapy*	n=174	n=11	n=11	n=8	n=10	n=84	n=26	n=23
**Monotherapy**								
**Triazoles**								
**FLC**	9 (5%)	2 (18%)	1 (9%)	1 (13%)	1 (10%)	1 (1%)	3 (12%)	0 (0%)
**ISA**	0 (0%)	0 (0%)	0 (0%)	0 (0%)	0 (0%)	0 (0%)	0 (0%)	0 (0%)
**ITC**	68 (39%)	0 (0%)	3 (27%)	4 (50%)	3 (30%)	48 (57%)	7 (27%)	3 (13%)
**KCZ**	6 (3%)	0 (0%)	0 (0%)	0 (0%)	2 (20%)	3 (4%)	1 (4%)	0 (0%)
**POS**	2 (1%)	1 (9%)	0 (0%)	0 (0%)	0 (0%)	1 (1%)	0 (0%)	0 (0%)
**VRC**	16 (9%)	4 (36%)	3 (27%)	1 (13%)	1 (10%)	7 (8%)	0 (0%)	0 (0%)
**Echinocandins**								
**CAS**	1 (1%)	0 (0%)	0 (0%)	1 (13%)	0 (0%)	0 (0%)	0 (0%)	0 (0%)
**MFG**	0 (0%)	0 (0%)	0 (0%)	0 (0%)	0 (0%)	0 (0%)	0 (0%)	0 (0%)
**Others**								
**AmB-DOC**	12 (7%)	1 (9%)	2 (18%)	0 (0%)	2 (20%)	7 (8%)	0 (0%)	0 (0%)
**L-AmB**	2 (%)	1 (9%)	0 (0%)	1 (13%)	0 (0%)	0 (0%)	0 (0%)	0 (0%)
**TBF**	10 (6%)	0 (0%)	0 (0%)	1 (13%)	0 (0%)	9 (11%)	0 (0%)	0 (0%)
**5-FC**	1 (1%)	0 (0%)	1 (9%)	0 (0%)	0 (0%)	0 (0%)	0 (0%)	0 (0%)
**KI**	2 (1%)	0 (0%)	0 (0%)	0 (0%)	0 (0%)	2 (2%)	0 (0%)	0 (0%)
**Combination therapy**								
**Azole + AmB-DOC**	10 (6%)	3 (27%)	3 (27%)	0 (0%)	3 (30%)	1 (1%)	0 (0%)	0 (0%)
**Azole + L-AmB**	4 (2%)	3 (27%)	1 (9%)	0 (0%)	0 (%)	0 (0%)	0 (0%)	0 (%)
**Azole + Echinocandin**	2 (1%)	0 (0%)	0 (0%)	2 (25%)	0 (0%)	0 (0%)	0 (0%)	0 (0%)
**Azole + KI**	1 (1%)	0 (0%)	0 (0%)	0 (0%)	0 (0%)	1 (1%)	0 (0%)	0 (0%)
**Azole + TBF**	14 (8%)	0 (0%)	1 (9%)	0 (0%)	1 (10%)	12 (14%)	0 (0%)	0 (0%)
**Azole+5-FC**	3 (2%)	1 (9%)	1 (9%)	0 (0%)	0 (0%)	1 (1%)	0 (0%)	0 (0%)
**Two Triazoles**	3 (2%)	0 (0%)	0 (0%)	0 (0%)	1 (10%)	0 (0%)	2 (8%)	0 (0%)
**AmB-DOC + Echinocandin**	1 (1%)	0 (0%)	0 (0%)	1 (13%)	0 (0%)	0 (0%)	0 (0%)	0 (0%)
**AmB-DOC + TBF**	1 (1%)	0 (0%)	0 (0%)	0 (0%)	0 (0%)	1 (1%)	0 (0%)	0 (0%)
**L-AmB + TBF**	1 (1%)	0 (0%)	0 (0%)	1 (13%)	0 (0%)	0 (0%)	0 (0%)	0 (0%)
**AmB-DOC + 5-FC**	2 (2%)	1 (9%)	0 (0%)	0 (0%)	0 (0%)	1 (1%)	0 (0%)	0 (0%)
**L-AmB + 5-FC**	1 (%)	1 (9%)	0 (0%)	0 (0%)	0 (0%)	0 (0%)	0 (0%)	0 (0%)
**NS + KI**	2 (1%)	0 (0%)	1 (9%)	0 (0%)	0 (0%)	1 (1%)	0 (0%)	0 (0%)
**Triple-Drug Combination**	3 (2%)	1 (9%)	1 (9%)	0 (0%)	0 (0%)	1 (1%)	0 (0%)	0 (0%)
**Four-Drug Combination**	0 (0%)	0 (0%)	0 (0%)	0 (0%)	0 (0%)	0 (0%)	0 (0%)	0 (0%)
**Non-Systematic Therapy**								
**Cryotherapy**	2 (1%)	0 (0%)	0 (0%)	0 (0%)	0 (0%)	2 (2%)	0 (0%)	0 (0%)
**Laser**	2 (1%)	0 (0%)	0 (0%)	0 (0%)	0 (0%)	2 (2%)	0 (0%)	0 (0%)
**Local Drug Infiltration**	58 (33%)	1 (9%)	1 (9%)	0 (0%)	4 (40%)	16 (19%)	20 (77%)	16 (70%)
**ALA-PDT**	2 (1%)	0 (0%)	0 (0%)	0 (0%)	0 (0%)	2 (2%)	0 (0%)	0 (0%)
**Surgery**	37 (21%)	4 (36%)	2 (18%)	0 (0%)	6 (60%)	15 (18%)	9 (35%)	1 (4%)
**Thermotherapy**	8 (5%)	0 (0%)	1 (9%)	0 (0%)	0 (0%)	7 (8%)	0 (0%)	0 (0%)
**Unknow**	19 (11%)	2 (18%)	1 (9%)	1 (13%)	0 (0%)	6 (7%)	2 (8%)	6 (26%)

FLC, fluconazole; ISA, isavuconazole; ITC, itraconazole; KCZ, ketoconazole; POS, posaconazole; VRC, voriconazole; CAS, caspofungin; MFG, micafungin; 5-FC, 5-fluorocytosine; AmB-DOC, amphotericin B deoxycholate; L-AmB, liposomal amphotericin B; TBF, terbinafine; ALA-PDT, 5-aminolevulinic acid-photodynamic therapy.

*Some patients were treated with more than one antifungal or combination of antifungal drugs in different periods.

Other treatments included surgeries and physical therapies. The operative rates were 60% (6 of 10), 36% (4 of 11), 35% (9 of 26), and 12% (15 of 84) respectively in the deep-local, CNS, corneal, and subcutaneous infections. The physical therapies contained thermotherapy, cryotherapy, laser, and 5-aminolevulinic acid-photodynamic therapy (ALA-PDT). Hyperthermia was used marginally in subcutaneous PHM (8%, 7 of 84) and disseminated PHM (9%, 1 of 11).

AmB-DOC/L-AmB and voriconazole were used most frequently in CNS, disseminated and pulmonary infections that improved or cured at the end of follow-up. The efficacy of L-AmB was better than AmB-DOC in some individual cases, whereas AmB-DOC was mainly used in China as we counted. The overall mortality rates of these three infections were 82% (9 of 11), 55% (6 of 11), and 75% (6 of 8) and their authentic mortality rates due to fungal infections were 55% (6 of 11), 36% (4 of 11) and 25% (2 of 8) respectively. The effective rates in the late stages were extremely low. Four patients with severe pulmonary infections all died despite attempts to use a variety of antifungal drugs including caspofungin. The cure rate of early surgical excision of localized brain cysts was 75% (3 of 4) and the effective rate was 100% (4 of 4). The subcutaneous PHM had an overall effective rate of 66% (56 of 85), and a cure rate of 45% (38 of 85). The subcutaneous nodules were resected in only 10 patients of whom the cure rate was 70% (7 of 10). In addition, among the 48 PHM patients who used a single antifungal agent–itraconazole at a certain stage, 11 cases had little effect and 24 cases were cured, so the effective rate was 73% (35 of 48) and the cure rate was 50% (24 of 48). The effective rates of keratitis and superficial PHM were 77% (20 of 26) and 70% (16 of 23). Their prognosis was favorable except for the permanent vision loss in a portion of keratitis.

### *In Vitro* Antifungal Susceptibility

In this study, 38 clinical fungal isolates from clinical specimens provided the minimum inhibitory concentration (MIC)/minimal effective concentration (MEC) values (**Supplementary Table 4**). We further linked the 16 dematiaceous fungal isolates’ MIC values to their clinical efficacy when a single triazole agent–itraconazole was used (**Supplementary Table 5**). At present, there is no clinical or epidemiological cut-off point for drug susceptibility of melanized fungi, so we reported the results based on the previous literature (Revankar and Sutton, 2010), MIC/MEC of ≤1 μg/ml was used as the index of potential sensitivity to most antifungal drugs to treat melanized molds, flucytosine (5-FC) (<50 μg/ml) excluded.

As a result, the drug resistance rate to fluconazole among the clinical dematiaceous fungi was the highest, accounting for 93% (25 of 27). The average MICs of echinocandins were higher than triazole drugs and had high variability *in vitro* activity. Most clinical strains were susceptible to terbinafine (89%, 16 of 18), voriconazole (86%, 18 of 21), and itraconazole (77%, 27 of 35). The average MICs of terbinafine were the lowest among all antifungal agents. AmB (56%, 18 of 32) was relatively less susceptible *in vitro*. Furthermore, the coincidence rate of itraconazole between MICs and clinical efficacy was 81% (13 of 16).

## Discussion

In the last 35 years, the quantity of phaeohyphomycosis was growing year by year, especially in southern China (Huang et al., 2019). The overall development of detection techniques of melanized fungi and more attention paid to this disease particularly contributed to the explosive growth. The widespread use of immunosuppressive agents, and improved microbiologic and gene testing techniques also made the incidence of PHM fast increase.

Melanized fungi are widely distributed in China. According to the sample research made in southwest China, there were at least 100 species of dark molds in the provinces of Guangxi, Guizhou, Hainan, Yunnan, and Chungking (Ma, 2018). There is probably underestimating of the disease in southwest China. The actual incidence of PHM may be higher in the less developed southern regions of China, such as Yunnan, Guizhou, and Hainan. No case has beenN reported till now which may be due to the lack of adequate awareness and the lack of advanced molecular methods which aid in identifying the agents of PHM, which might be the cause of underreporting of cases in these less developed areas. It may be the truth not just in China, but all around the world, especially the disadvantaged tropical countries.

The growing body of melanotic fungi pathogenesis is being gradually discovered and refined nowadays (Eisenman et al., 2020). One of the major virulence factors is the cell-wall melanin, which was once thought to prevent the fungi from an oxidative explosion inside phagocytes. However, other mechanisms of melanin have been elucidated such as impacting host cell signaling (Shi et al., 2019), blocking the autophagy pathway LC3-associated phagocytosis (LAP) (Andrianaki et al., 2018), and interfering with recognition of the fungal cells through an identified receptor called MelLec that is diffusely expressed in epithelial tissues (Stappers et al., 2018). Furthermore, a recent founding has verified that some fungi can get resistant to antifungals by increasing melanin production (Heidrich et al., 2021). On the other hand, transcriptomics research on melanotic fungal genomes is recognized as cutting-edge and promising. Several studies made efforts to find genetic profiles by comparing rare human neurotropic pathogens with other known black fungi and made transcriptomic analysis of an albino mutant comparing its melanized strains, which showed that not only some highly expressed melanin genes but also some differentially expressed genes (DEGs) which associated with survival pathway, cell growth, and metabolic pathways (Chen et al., 2014; Li et al., 2016; Bombassaro et al., 2020).

In our research, we find that the most common risk factors are trauma. Traumatic implantation mainly comes from the departments of agriculture, fishery, forestry, mining, construction, and sanitation. The above occupations are difficult to avoid the daily invisible contamination injuries so more occupational protections are needed. The other major factors that reduced immune functions include malnutrition, tumors, and kidney transplantations. Under the background of the global COVID-19 pandemic, Simin Laiq was the first to report a case of *Fonsecaea*-associated cerebral PHM in a 73 years old Omani diabetic lady who presented headache and visual disturbance 6 weeks after recovery from COVID-19 pneumonia (Laiq et al., 2022). Actually, treatment with steroids and exacerbation of diabetes during COVID-19 infection may lead patients to be immunocompromised and more susceptible to fungal infections. Notably, with the popularization of genetic testing technology, inherited CARD9 deficiency was found in 12 patients from China in recent 5 years. This group of patients usually were tricky to cure and had a long course of the disease, indicating that anti-fungal immunodeficiency caused by the CARD9 gene mutation was one of the pathogenic factors. A large portion of PHM patients without known immunodeficiencies may have the potential mutations of the CARD9 gene if the gene test could be done in the past (Huang et al., 2019).

There is no uniform approach for the treatment of PHM. We advocate early diagnosis and treatment of PHM. Surgery is the most important for cerebral cysts, but may also be critical for other forms of local lesions such as subcutaneous nodules, keratitis, bone and joint infection, and catheter removal. There is also no standard approach when it comes to antifungal drugs. Global guidelines for rare mold infections proposed by the European Confederation of Medical Mycology (ECMM) union with the International Society for Human and Animal Mycology and the American Society for Microbiology (ISHAM) in 2021 strongly recommend itraconazole or voriconazole as the first-line treatment for superficial PHM and subcutaneous PHM (Hoenigl et al., 2021). When referring to disseminated PHM involved CNS and local-deep organs, posaconazole and voriconazole plus L-AmB are moderately recommended as first-line drug treatments. Echinocandins and terbinafine may be combined with them but are not recommended to be applied solely. In 2014, the guidelines for PHM made by the experts of ESCMID and ECMM showed the recommendations that topical 5% natamycin and amphotericin B (0.15–0.3%) were strongly regarded as the first-line therapy for most keratitis. Oral triazoles with surgery or even an intracorneal injection of voriconazole (1%) as moderately recommended salvage therapy for severe and refractory corneal infections. When it comes to cerebral infections, first and foremost is complete excision of brain abscesses (grade AII) rather than only partial excision or aspiration. When surgery is not possible, a triazole combined with an echinocandin plus flucytosine is moderately recommended to be the first-line therapy (grade BIII). Voriconazole and posaconazole (grade CII) are marginally recommended while AmB-DOC therapy alone is in the lowest recommended grade which is against use (Chowdhary et al., 2014).

Isavuconazole and ravuconazole are two new triazoles antifungal agents. In 2019 a study using these two agents to test their *in vitro* susceptibility of a broad spectrum of dematiaceous fungi showed better antifungal activity than itraconazole, voriconazole, and posaconazole, especially against *Bipolaris spicifera* and *Veronaea botryosa* (Zheng et al., 2020). And also, in 2022 a research project verified that ravuconazole could be recognized as a promising drug candidate for the treatment of eumycetoma and is at present being examined in a randomized, double-blinded clinical trial for mycetoma. In the clinical trial, the efficacy of weekly treatment of fosravuconazole at 200 or 300 mg is compared to the daily of itraconazole at 400 mg in mycetoma patients (Lim et al., 2022). However, the clinical safety and efficacy of these two antifungal agents in the treatment of PHM have not been reported. Detailed case reports of success and failure experiences still need to be explored and accumulated in the later stage. Thus, it can be seen, that antifungal susceptibility test *in vitro* plays a critical role in the management of fungal infections and the selection of optimal drug therapy.

When it comes to morbidity, subcutaneous PHM has the highest incidence. We found that the severity grades of lesion have a close correlation with the pathogens’ virulence and the patient’s immune status, which may predict the patient’s clinical outcome. In our statistics, severe rashes are much more pleomorphic, covering almost all types of rashes, including scars and sinus. Areas rich in capillaries such as the head and face are mostly affected accounting for 16% in severe cases. It’s easy to cause disfigurement, disability, and hematogenous spread. The data suggested that the grade of the lesion has a level of significant correlation with the course of the disease (P=0.098) and the effective rate (P=0.08). But there’s no significant variation in cure rate (P=2.06), or death rate (P=2.57) between mild, moderate, and severe rashes. We hypothesized that the more severe rash was related to the stronger pathogen’s virulence and the lower patient’s immune status. The worse rashes have higher dark fungal loads, and these strains may become resistant to antifungal agents for long-term treatment. Notwithstanding, the follow-up periods can be recorded from the whole literature in this study ranging from 4 weeks to 4 years. Many patients are lost to follow-up because there are no standard cure criteria for the PHM. Lots of people who responded to therapy did not have a prolonged follow-up, and therefore the final cure rates of severe PHM may be much lower and the death rates may be much higher in reality.

Overall, with the increasing attention paid to this kind of infection, the continuous improvement of diagnostic methods, the iterative updating of new drugs with better efficacy, and the deepening of clinical research, it is expected to realize the early diagnosis of dark fungal infections with high sensitivity and specificity and more targeted individual treatment.

## Conclusion

A total of 174 phaeohyphomycosis patients have been reported in China in the past 35 years. Among these CNS, disseminated and pulmonary types have the highest mortality. The subcutaneous type is the most common, and the corneal type ranks second. Most cases are concentrated in southeast China. The early clinical manifestations of PHM are nonspecific and its misdiagnosis rate is as high as 74%. Nearly 30% of invasive infections of PHM start from persistent and recurrent lesions. Subcutaneous lesions can be divided into three grades: mild (15, 18%), moderate (39, 46%), and severe (31, 36%). We discover that there’re no significant variations in cure rate, or death rate between three grades of lesions. But patients with severe rashes have much lower effective rates and the immune status of this population is relatively weaker. Treatment of PHM remains tricky. Our researchers need to conduct more studies: 1, To fully understand the melanin and other virulence factors in dark fungi. 2, To find more additional susceptible gene mutations in patients. 3, And to explore the possibility of discovering more effective and promising agents.

## Author Contributions

All authors listed have made a substantial, direct, and intellectual contribution to the work, and approved it for publication.

## Funding

This study was funded by the National Science and Technology Infrastructure of China (Project No. National Pathogen Resource Center–NPRC–32) and the National Science and Technology Major Project (2018ZX10734404).

## Conflict of Interest

The authors declare that the research was conducted in the absence of any commercial or financial relationships that could be construed as a potential conflict of interest.

## Publisher’s Note

All claims expressed in this article are solely those of the authors and do not necessarily represent those of their affiliated organizations, or those of the publisher, the editors and the reviewers. Any product that may be evaluated in this article, or claim that may be made by its manufacturer, is not guaranteed or endorsed by the publisher.
