# Supplementary material for: Phaeohyphomycosis in China
Source: Front Cell Infect Microbiol. 2022 Jun 13;12:895329. doi: 10.3389/fcimb.2022.895329 (PMC9235401; doi:10.3389/fcimb.2022.895329)
Supplement: Supplementary file 1 [file DataSheet_1.docx]

**List of All Topics:**

**1. Basis and Rationale of the Classification (Table 1)**

**2. Fungal Genera Isolated From 174 Patients with Phaeohyphomycosis in China (Table 2)**

**3. Outcomes of 174 Patients with Phaeohyphomycosis in China (Table 3)**

**4. MIC (µg/mL) for 38 Dematiaceous Fungal Isolates of PHMs in China (Table 4)**

**5. MICs(µg/mL) and Clinical Efficacy of Itraconazole for 16 PHMs’ Fungal Isolates in China (Table 5)**

**6. Reference**

**1. Basis and Rationale of the Classification**

In 1983, McGinnis firstly defined and classified PHMs.^1^ With the increasing of PHMs cases, Sanjay G. Revankar made a new classification in 2010 and divided PHM into five types: (1) superficial infection, (2) deep local infection, (3) pulmonary infection, (4) central nervous system infection, and (5) disseminated infection.^2^ (Table 1)

**Table 1.** Infection types of the phaeohyphomycosis according to Sanjay G. Revankar’s review

| Infection types | Subtypes |
| --- | --- |
| central nervous system infection | - |
| disseminated infection | - |
| pulmonary infection | - |
| deep local infection | endophthalmitis |
|  | acute invasive rhinosinusitis |
|  | hepatic infection |
|  | bone and joint infection |
|  | peritonitis |
|  | pleuritis |
|  | lymphnoditis |
|  | subcutaneous lesion |
|  | keratitis |
| superficial infection | tinea nigra |
|  | cutaneous |
|  | onychomycosis |

**2. Fungal Genera Isolated From 174 Patients with Phaeohyphomycosis in China**

**Table 2.** Fungal Genera Isolated From 174 Patients with Phaeohyphomycosis in China

|  |  | Total | CNS | Disseminated | Pulmonary | Local-Deep | Subcutaneous | Keratitis | Superficial |
| --- | --- | --- | --- | --- | --- | --- | --- | --- | --- |
| No. | Genus | 174 | n=11 | n=11 | n=8 | n=10 | n=85 | n=26 | n=23 |
| 1 | *Alternaria alternata* | 5 |  |  |  | 1 | 4 |  |  |
| 2 | *Arthrinium phaeospermum* | 4 |  |  |  |  | 4 |  |  |
| 3 | *Aureobasidium pullulans* | 3 |  | 1 |  |  | 1 |  | 1 |
| 4 | *Bipolaris oryzae* | 1 |  |  |  |  |  | 1 |  |
| 5 | *Bipolaris spicifera* | 2 | 1 |  |  |  | 1 |  |  |
| 6 | *Chaetomium atrobrunneum* | 7 |  |  | 1 |  | 1 | 5 |  |
| 7 | *Chaetomium globosum* | 2 |  |  |  |  | 1 |  | 1 |
| 8 | *Chaetomium murorum* | 1 |  |  |  |  | 1 |  |  |
| 9 | *Cladophialophora bantiana* | 3 | 2 |  |  |  | 1 |  |  |
| 10 | *Cladosporium cladosporioides* | 6 |  |  | 1 |  | 4 |  | 1 |
| 11 | *Cladosporium herbarum* | 1 |  |  |  |  | 1 |  |  |
| 12 | *Cladosporium sphaerospermum* | 2 | 1 |  |  |  | 1 |  |  |
| 13 | *Colletotrichum dematium* | 1 |  |  |  |  |  | 1 |  |
| 14 | *Colletotrichum fructicola* | 2 |  |  |  |  |  | 2 |  |
| 15 | *Colletotrichum fusiforme* | 1 |  |  |  |  |  | 1 |  |
| 16 | *Colletotrichum tropicale* | 3 |  |  |  |  |  | 3 |  |
| 17 | *Colletotrichum truncatum* | 1 |  |  |  |  |  | 1 |  |
| 18 | *Corynespora cassiicola* | 4 |  |  |  |  | 4 |  |  |
| 19 | *Curvularia clavata* | 2 |  |  |  |  | 2 |  |  |
| 20 | *Curvularia lunata* | 5 |  |  |  |  |  | 5 |  |
| 21 | *Exophiala asiatica* | 1 |  | 1 |  |  |  |  |  |
| 22 | *Exophiala bergeri* | 1 |  |  |  |  |  |  | 1 |
| 23 | *Exophiala dermatitidis* | 10 | 4 | 3 |  | 1 | 1 | 1 |  |
| 24 | *Exophiala hongkongensis* | 1 |  |  |  |  |  |  | 1 |
| 25 | *Exophiala jeanselmei* | 18 |  | 1 | 1 | 1 | 15 |  |  |
| 26 | *Exophiala lecanii-corni* | 2 |  |  |  |  | 1 |  | 1 |
| 27 | *Exophiala oligosperma* | 6 |  |  | 1 |  | 2 |  | 3 |
| 28 | *Exophiala spinifera* | 8 |  | 2 |  |  | 6 |  |  |
| 29 | *Exophiala xenobiotica* | 1 |  |  |  | 1 |  |  |  |
| 30 | *Exserohilum rostratum* | 7 |  |  |  |  | 3 | 3 | 1 |
| 31 | *Hongkongmyces pedis* | 1 |  |  |  |  | 1 |  |  |
| 32 | *Hongkongmyces snookiorum* | 1 |  |  |  |  | 1 |  |  |
| 33 | *Hortaea werneckii* | 10 |  |  |  |  |  |  | 10 |
| 34 | *Knufia epidermidis* | 1 |  |  |  |  |  |  | 1 |
| 35 | *Lasiodiplodia theobromae* | 1 |  |  | 1 |  |  |  |  |
| 36 | *Medicopsis romeroi* | 2 |  |  |  |  | 2 |  |  |
| 37 | *Microsphaeropsis arundinis* | 3 |  | 1 |  |  | 2 |  |  |
| 38 | *Neocucurbitaria unguis-hominis* | 1 |  |  |  |  | 1 |  |  |
| 39 | *Neoscytalidium dimidiatum* | 2 |  |  |  |  | 2 |  |  |
| 40 | *Nigrograna mackinnonii* | 1 |  |  |  |  | 1 |  |  |
| 41 | *Nigrospora sphaerica* | 1 |  |  |  |  |  |  | 1 |
| 42 | *Ochroconis musae* | 1 |  |  |  |  | 1 |  |  |
| 43 | *Ochroconis tshawytschae* | 1 |  |  |  |  | 1 |  |  |
| 44 | *Pallidocercospora crystallina* | 1 |  |  |  |  | 1 |  |  |
| 45 | *Phaeoacremonium parasiticum* | 4 |  |  | 1 | 2 |  |  | 1 |
| 46 | *Phialemoniopsis hongkongensis* | 1 |  |  |  |  | 1 |  |  |
| 47 | *Phialophora americana* | 1 |  |  |  |  | 1 |  |  |
| 48 | *Phialophora macrospora* | 2 |  |  |  |  | 2 |  |  |
| 49 | *Phialophora tarda* | 3 |  | 1 |  |  | 2 |  |  |
| 50 | *Phialophora verrucosa* | 1 |  |  |  | 1 |  |  |  |
| 51 | *Pleurostoma hongkongense* | 1 |  |  |  | 1 |  |  |  |
| 52 | *Rhinocladiella basitona* | 2 |  |  |  | 1 | 1 |  |  |
| 53 | *Roussoella percutanea* | 1 |  |  |  |  | 1 |  |  |
| 54 | *Veronaea botryosa* | 5 |  |  |  |  | 5 |  |  |
| 55 | *Verruconis gallopava* | 2 |  | 1 | 1 |  |  |  |  |
|  | unknown | 13 | 3 | 0 | 1 | 1 | 5 | 3 | 0 |

**3. Outcomes of 174 Patients with Phaeohyphomycosis in China**

**Table 3.** Outcomes of 174 Patients with Phaeohyphomycosis in China

| No. (Ratio%) | Total | CNS | Disseminated | Pulmonary | Deep-local | Subcutaneous | Keratitis | Superficial |
| --- | --- | --- | --- | --- | --- | --- | --- | --- |
| Outcome | n=174 | n=11 | n=11 | n=8 | n=10 | n=85 | n=26 | n=23 |
| 30-Day Response |  |  |  |  |  |  |  |  |
| Complete | 30 (17%) | 0 (0%) | 0 (0%) | 0 (0%) | 3 (30%) | 8 (9%) | 8 (31%) | 11 (48%) |
| Partial | 92 (53%) | 5 (45%) | 7 (64%) | 4 (50%) | 6 (60%) | 51 (60%) | 15 (58%) | 4 (17%) |
| Failure | 29 (17%) | 6 (55%) | 3 (27%) | 3 (38%) | 1 (10%) | 13 (15%) | 2 (8%) | 1 (4%) |
| End-of-Follow-up Response |  |  |  |  |  |  |  |  |
| Complete | 82 (47%) | 4 (36%) | 2 (18%) | 3 (38%) | 8 (80%) | 38 (45%) | 12 (46%) | 15 (65%) |
| Partial | 33 (19%) | 1 (9%) | 1 (9%) | 0 (0%) | 2 (20%) | 18 (21%) | 8 (31%) | 1 (4%) |
| Failure | 32 (18%) | 6 (55%) | 7 (64%) | 4 (50%) | 0 (0%) | 16 (19%) | 0 (0%) | 1 (4%) |
| Mortality |  |  |  |  |  |  |  |  |
| ≤ 30 d | 5 (3%) | 1 (9%) | 1 (9%) | 1 (13%) | 0 (0%) | 2 (2%) | 0 (0%) | 0 (0%) |
| End of Follow-up | 12 (7%) | 2 (18%) | 1 (9%) | 3 (38%) | 0 (0%) | 6 (7%) | 0 (0%) | 0 (0%) |
| Due to Fungal Infection | 12 (7%) | 6 (55%) | 4 (36%) | 2 (25%) | 0 (0%) | 0 (0%) | 0 (0%) | 0 (0%) |
| Follow-up, Median (Range), d | 150  (1-4745) | 240  (13-1095) | 180  (30-4745) | 43  (1-1460) | 243  (14-1095) | 180  (14-1643) | 51  (14-730) | 150  (14-365) |
| Unknow | 20 (11%) | 0 (0%) | 1 (9%) | 1 (13%) | 0 (0%) | 12 (14%) | 0 (0%) | 6 (26%) |

**4. MIC (µg/mL) for 38 Dematiaceous Fungal Isolates of PHMs in China**

Table 4. MIC (µg/mL) for 38 Dematiaceous Fungal Isolates of PHMs in China

|  | MICs/MECs (ug/mL) | | | | | | | | | | | | |
| --- | --- | --- | --- | --- | --- | --- | --- | --- | --- | --- | --- | --- | --- |
|  |  | Triazoles | | | | | | Echinocandins | | | Others | | |
| Species (Year, Author) | FLC | ISA | ITC | KCZ | POS | RAV | VRC | AFG | CAS | MFG | 5FC | AMB | TBF |
| CNS | | | | | | | | | | | | | |
| *Exophiala dermatitidis (2009, Chang XZ)* | 16 | – | 1 |  | – | – | – | – | – | – | 64 | 0.5 | 0.03 |
| Disseminated |  |  |  |  |  |  |  |  |  |  |  |  |  |
| *Exophiala asiatica (2009 Dong ML)* | 16/64 | – | 0.25/0.5 | 0.25/0.25 | – | – | – | – | – | – | 4/4 | 0.25/1 | 0.03/0.03 |
| *Exophiala dermatitidis (2005, Tseng PH)* | 48 | – | – |  | – | – | – | – | – | – | – | 0.19 | – |
| *Phialophora tarda (2013, Gao LJ)* | ≥64 | – | 1 |  | 0.125 | – | 1 | – | 2 | 1 | ≥32 | 4 | 0.006 |
| Pneumonia | | | | | | | | | | | | | |
| *Alternaria spp. (2010, Xu XL))* | 1 | – | 0.125 |  | – | – | – | – | – | – | ≤4 | 1 | – |
| *Chaetomium atrobrunneum (2016, Wang H)* | 16 | – | 0.25 |  | – | – | – | – | – | – | – | 0.032 | – |
| *Cladosporium cladosporioides (2018, Liu SJ)* | – | – | – |  | – | – | 0.75 | – | – | – | – | 0.064 | – |
| Deep–Local | | | | | | | | | | | | | |
| *Exophiala dermatitidis (2002, Liou JM)* | 16 | – | 0.25 |  | – | – | – | – | – | – | – | 0.032 | – |
| *Phaeoacremonium parasiticum (2014, Wang Q)* | – | – | 32 |  | – | – | 0.25 | – | – | – | – | 32 | – |
| *Phialophora verrucose (2010, Shi YS)* | 32 | – | 0.5 | 16 | – | – | 1 | – | – | – | 32 | 0.5 | – |
| *Rhinocladiella basitona (2015, Liu MY)* | 64 | – | 0.5 | 1 | – | – | 0.25 | – | – | – | – | 0.5 | – |
| *Pleurostoma hongkongense (2021, Tsang CC)* | ＞16 | ＞16 | ＞8 |  | ＞8 | – | ＞16 | ＞16 | 16 | ＞16 | ＞16 | 0.5 | – |
| Subcutaneous | | | | | | | | | | | | | |
| *Arthrinium phaeospermum (2005, Lv GX)* | ＞64 | – | ＞64 | 4 | – | – | – | – | – | – | – | – | 0.125 |
| *Arthrinium phaeospermum (2016, Chen XW)* | – | – | – | – | 0.5 | 0.03125 | 0.25 | – | 0.5 | 0.25 | – | – | 2 |
| *Arthrinium phaeospermum (2016, Hu SQ)* | ＞64 | – | 0.5 | – | – | – | – | – | – | – | – | 0.5 | 1 |
| *Chaetomium atrobrunneum (2010, Zhang H)* | 64 | – | 0.04 | 0.06 | – | – | – | – | – | – | ＞256 | 1.12 | – |
| *Chaetomium globosum (2006, Jin Y)* | ＞64 | – | 0.5 | – | – | – | – | – | – | – | – | 4 | 8 |
| *Cladosporium cladosporioides (2016, Zhou YB)* | 64 | – | 0.125 | – | – | – | 0.5 | – | – | – | ≤4 | 0.5 | – |
| *Corynespora cassiicola (2011, Lv GX)* | 8 | – | 8 | – | – | – | – | – | – | – | – | – | 0.125 |
| *Corynespora cassiicola (2018, Wang XW)* | – | – | 4 | – | – | – | 0.25 | – | 16 | – | – | 2 | 0.125 |
| *Exophiala dermatitidis (2016, Chen M)* | 0.125 | – | 0.5 | – | – | – | 4 | 4 | – | 4 | – | 4 | 0.25 |
| *Exophiala jeanselmei (2009, Zhou CJ)* | 128 | – | 0.25 | – | – | – | 0.125 | – | – | – | <4 | 4 | – |
| *Exophiala jeanselmei (2012, Zhou XY)* | 32 | – | 0.2 | – | – | – | 0.094 | – | 32 | – | 32 | 1 | – |
| *Exophiala jeanselmei (2002, Liou JM)* | 16 | – | 0.25 | – | – | – | – | – | – | – | – | 0.032 | – |
| *Exophiala oligosperma (2018, Hong Y)* | ＞32 | – | 0.5 | – | – | – | – | – | – | – | – | – | 0.03 |
| *Exophiala spinifera (2018, Wang XW)* | – | – | 0.5 | – | – | – | 0.25 | – | 16 | – | – | 2 | 0.25 |
| *Exophiala spinifera (2015, Wang L)* | – | – | 0.25 | 0.25 | – | – | – | – | ＞32 | – | ＞32 | 0.25 | – |
| *Hongkongmyces pedis (2015, Tsang CC)* | 8 | – | 0.06 | – | 0.06 | – | 0.25 | – | – | – | 8 | 4 | – |
| *Hongkongmyces snookiorum (2020, Deng LQ)* | – | – | 0.012 | – | – | – | 0.008 | – | – | – | – | 4 | – |
| *Ochroconis musae (2018, Wang XW)* | – | – | 2 | – | – | – | 4 | – | 16 | – | – | 4 | 0.002 |
| *Ochroconis tshawytschae (2012, Lv GX))* | 64 | – | 0.5 | 2 | – | – | 0.125 | – | 0.25 | – | – | 4 | – |
| *Phialemoniopsis hongkongensis (2014, Tsang CC)* | 16 | – | 0.5 | – | 0.5 | – | 0.12 | – | – | – | ＞64 | 1 | – |
| *Rhinocladiella basitona (2013, Cai Q)* | ＞64 | – | 0.5 | 1 | – | – | 1 | – | – | 2 | – | – | 0.03 |
| *Veronaea botryose (2011, Sang H)* | ＞64 | – | 2 | 2 | – | – | – | – | – | – | – | 1 | 1 |
| *Veronaea botryose (2006, Chen YT)* | – | – | ＞256 | – | – | – | – | – | – | – | – | ＞32 | – |
| Keratitis | | | | | | | | | | | | | |
| *Alternaria arborescens (2009, Li JZ)* | ≤8 | – | ≤0.125 | ≤1 | – | – | ≤1 | – | – | – | ≥32 | ≤1 | ≤1 |
| *Bipolaris oryzae (2015, Wang LX)* | – | – | 0.25 |  | – | – | 0.06 | – | – | – | – | 2 | 0.125 |
| Onychomycosis | | | | | | | | | | | | | |
| *Chaetomium globosum (2016, Shi DM)* |  |  | 0.125 | 0.25 |  |  |  |  |  |  |  |  | 0.5 |

MIC, Minimal inhibitory concentration; MEC, Minimal effective concentration; FLC, fluconazole; ISA, isavuconazole; ITC, itraconazole; KCZ, ketoconazole; POS, posaconazole; RAV, ravuconazole; VRC, voriconazole; AFG, anidulafungin; CAS, caspofungin; MFG, micafungin; 5FC, 5-fluorocytosine; AMB, amphotericin B; TBF, terbinafine.

**5. MICs(µg/mL) and Clinical Efficacy of Itraconazole for 16 PHMs’ Fungal Isolates in China**

Table 5. MICs(µg/mL) and Clinical Efficacy of Itraconazole for 16 PHMs’ Fungal Isolates in China

| Species (Year, Author) | Sex/age | Infection Site | Underlying Condition | MIC(μg/ml) | Clinical Effect |
| --- | --- | --- | --- | --- | --- |
| *Cladosporium cladosporioides (2018, Liu SJ)* | F/52 y | Lung | TB | Sensitive | Cured |
| *Chaetomium atrobrunneum (2010, Zhang H)* | M/2 y | Subcutaneous | None | 0·04 | Cured |
| *Arthrinium phaeospermum (2016, Chen XW)* | F/29 y | Subcutaneous | None | ＞64 | Cured |
| *Arthrinium phaeospermum (2016, Hu SQ)* | F/59 y | Subcutaneous | Trauma | 0·5 | Cured |
| *Cladosporium cladosporioides (2016, Zhou YB)* | M/21 y | Subcutaneous | None | 0.125 | Cured |
| *Exophiala dermatitidis (2016, Chen M)* | M/78 y | Subcutaneous | None | 0·5 | Improved |
| *Exophiala jeanselmei (2009, Zhou CJ)* | M/59 y | Subcutaneous | None | 0.25 | Cured |
| *Exophiala oligosperma (2018, Hong Y)* | F/37 y | Subcutaneous | None | 0·5 | Cured |
| *Exophiala spinifera (2015, Wang L)* | F/21 y | Subcutaneous | Pregnancy | 0.25 | Cured |
| *Ochroconis musae (2018, Wang XW)* | F/53 y | Subcutaneous | CARD9 Deficiency | 2 | Aggravation |
| *Phialemoniopsis hongkongensis (2014, Tsang CC)* | M/55 y | Subcutaneous | Trauma, PHBC, TB, AS | 0.5 | Aggravation |
| *Veronaea botryose (2011, Sang H)* | F/16 y | Subcutaneous | None | 2 | Improved |
| *Veronaea botryose (2006, Chen YT)* | M/76 y | Subcutaneous | DM, Cushing’s syndrome | ＞256 | Aggravation |
| *Alternaria arborescens (2002, Liu WD)* | F/62 y | Corneal | Trauma | Sensitive | Cured |
| *Aureobasidium pullulans (2016, Chen WT)* | M/46 y | Cutaneous | None | 0.25 | Improved |
| *Chaetomium globosum (2016, Shi DM)* | F/46 y | Nail | Trauma | 0.125 | Cured |

F, female; M, male; CARD9, caspase recruitment domain-containing protein 9; MIC, Minimal inhibitory concentration;

TB, tuberculosis; PHBC, post hepatitis B cirrhosis; AS, ankylosing spondylitis; DM, diabetes.

**6. Reference:**

1. McGinnis, M. R. (1983) Chromoblastomycosis and phaeohyphomycosis: new concepts, diagnosis, and mycology. *J Am Acad Dermatol*. 8(1), 1–16. doi: 10.1016/s0190-9622(83)70001-0
2. Revankar, S. G., Sutton, D. A. (2010) Melanized Fungi in Human Disease. *Clin Microbiol Rev*. 23, 884–928. doi: 10.1128/CMR.00019-10
